# Surface-Enhanced Raman Spectroscopy Assisted by Radical Capturer for Tracking of Plasmon-Driven Redox Reaction

**DOI:** 10.1038/srep30193

**Published:** 2016-07-22

**Authors:** Xuefeng Yan, Lingzhi Wang, Xianjun Tan, Baozhu Tian, Jinlong Zhang

**Affiliations:** 1 Key Laboratory for Advanced Materials and Institute of Fine Chemicals, East China University of Science and Technology, 130 Meilong Road, Shanghai 200237, P. R. China

## Abstract

The deep understanding about the photocatalytic reaction induced by the surface plasmon resonance (SPR) effect is desirable but remains a considerable challenge due to the ultrafast relaxation of hole-electron exciton from SPR process and a lack of an efficient monitoring system. Here, using the p-aminothiophenol (PATP) oxidation SPR-catalyzed by Ag nanoparticle as a model reaction, a radical-capturer-assisted surface-enhanced Raman spectroscopy (SERS) has been used as an *in-situ* tracking technique to explore the primary active species determining the reaction path. Hole is revealed to be directly responsible for the oxidation of PATP to p, p′-dimercaptoazobenzene (4, 4′-DMAB) and O_2_ functions as an electron capturer to form isolated hole. The oxidation degree of PATP can be further enhanced through a joint utilization of electron capturers of AgNO_3_ and atmospheric O_2_, producing p-nitrothiophenol (PNTP) within 10 s due to the improved hole-electron separation efficiency.

Decay of surface plasmon to hot carriers has found wide applications in energy conversion, photocatalysis and photodetection^1^,^2^. Among them, surface plasmonic resonance (SPR)-catalysed reactions have attracted increasing attention due to their green and convenient process^3^,^4^. Moreover, compared with the thermocatalysis, the SPR-catalysis often results in a unique selectivity^5^,^6^,^7^. However, due to the rapid relaxation process of hot carriers (hole and electron) on a short timescale ranging from femtosecond to picosecond, it is still hard to flexibly control the path/degree of SPR-catalysed reactions^8^,^9^,^10^.

Deep and accurate understanding about the mechanism of SPR-catalysed reaction is highly essential for designing an efficient reaction system, which requires a sensitive and *in-situ* analysis strategy. Thanks for the same origin of SPR-catalysed reaction and surface-enhanced Raman spectroscopy (SERS) with an ultrahigh sensitivity up to single-molecule level, SERS is allowed to *in-situ* monitor the SPR-catalysed reaction process through providing fingerprint spectra of surface species^11^,^12^,^13^,^14^,^15^,^16^. Some theoretical and indirect experiment proofs from SERS have assumed the oscillated hot electrons and molecular O_2_ are responsible for the SPR-catalysed redox reactions^17^,^18^. However, the active species determining the primary reaction step have still not been determined due to a lack of direct experimental proof.

Here, *in-situ* SERS technique aided with radical capturers for hot hole, electron and secondary radicals (•OH and •O_2_^−^), generated from the plasmon decay of Ag nanoparticles (NPs), has unprecedentedly been used to determine the active species by adopting the oxidation of p-aminothiophenol (PATP) as a model reaction^19^,^20^,^21^,^22^. Hole is revealed to be directly responsible for the oxidation of PATP to p, p′-dimercaptoazobenzene (4, 4′-DMAB)^23^,^24^. Chu prepared Ag/TiO_2_ system to investigate this reaction. The result shows that hot electrons generating from plasmonic decay would transfer to the conduction band of TiO_2_, which enables the holes to trigger the immediate conversion^25^. The role of O_2_ is determined as an electron capturer to produce isolated holes. PATP can be further oxidized to p-nitrothiophenol (PNTP) within 10 s through a joint utilization of electron capturers of AgNO_3_ and atmospheric O_2_^26^,^27^. To the best of our knowledge, this is the first time to unambiguously reveal the active species for the SPR-catalysed redox reaction through the *in-situ* SERS technique.

## Result and Discussion

**Figure 1:**
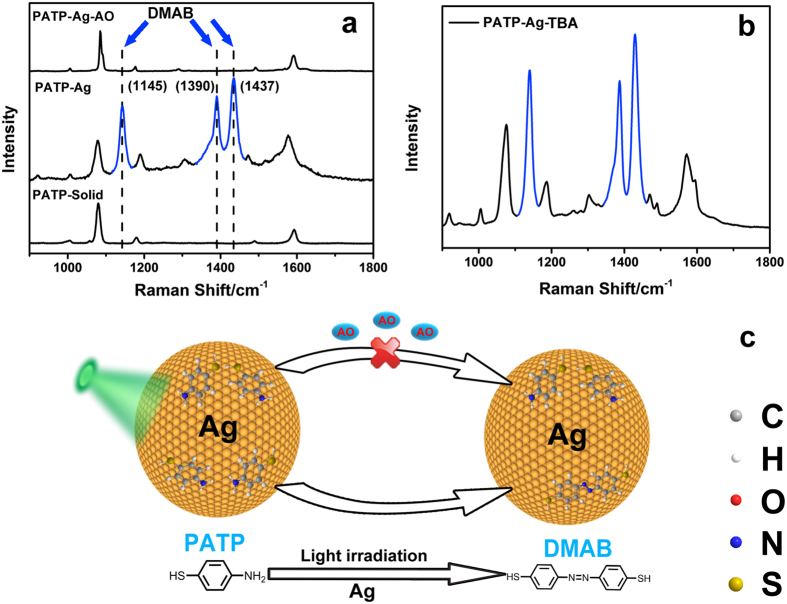
(**a**) Raman signal of PATP solid (black line), SERS signals of PATP on Ag nanoparticle layer in the absence and presence of AO; (**b**) SERS signal of PATP on assembly Ag nanoparticle layer in the presence of TBA; (**c**) Schematic diagram of plasmonic reactions on Ag layer in the presence of AO or TBA. Laser wavelength, 532 nm; Power, 2.5 mW; Integration time, 10 s.

Assembled Ag nanoparticle layer spin-coated from 50 nm of Ag NPs on a glass slide is used as plasmon-active substrate both for SERS analysis and catalysis reaction, which shows a wide plasmon resonance absorption band centred at 450 nm (Figures S1–S4). For assembled Ag nanoparticle layer adsorbed with PATP (Ag-PATP), Ag NPs and PATP are premixed before spin-coating. As shown in Fig. 1a, the SERS signal is collected under the irradiation of a 532 nm laser within 10 s. Compared with the Raman signal of PATP on the glass slide, three new peaks (blue line) assigned to the bend vibration of CH (β(CH)) at 1145 cm^−1^ and the stretching vibration of N = N (ν(N = N)) at 1390 and 1437 cm^−1^ are observed, implying PATP is oxidized to 4, 4′-DMAB as driven by the SPR effect of Ag nanoparticle layer^28^,^29^,^30^,^31^,^32^,^33^,^34^. All the three strongly enhanced peaks of DMAB represent symmetric a_g_ vibrational modes, strongly indicating the formation of DMAB from PATP through the N = N bond. These peaks are assigned to the a_g12_, a_g16_, and a_g17_ symmetric vibrational modes, respectively^35^,^36^. However, it is found that when ammonium oxalate (AO), a commonly used hole capturer is present, the reaction from PATP to 4, 4′-DMAB is completely quenched since no signal from 4, 4′-DMAB is observed. This result indicates the capturer-assisted strategy actually allows SERS to *in-situ* explore the reaction process in spite of the ultrafast relaxation process of hot carriers. Since both hole and its secondary radical •OH are strong oxidants, it still cannot be distinguished that PATP is oxidized by hole or •OH. Therefore, t-butanol (TBA) as the capturer for •OH is further adopted to understand its influence on the reaction^37^. However, strong peaks attributed to 4, 4′-DMAB is still observed with preserved intensity, suggesting the reaction from PATP to 4, 4′-DMAB is not altered by •OH (Fig. 1b). Therefore, it is undoubted that the oxidation of PATP is directly related to the hole decayed from the surface plasmon resonance of Ag nanoparticle layer. To get the original spectrum of PATP, it also can be detected on the Ag substrate by the irradiation of 785 nm on lower laser powers such as 0.50 μW and 0.25 mW (Figure S5).

**Figure 2:**
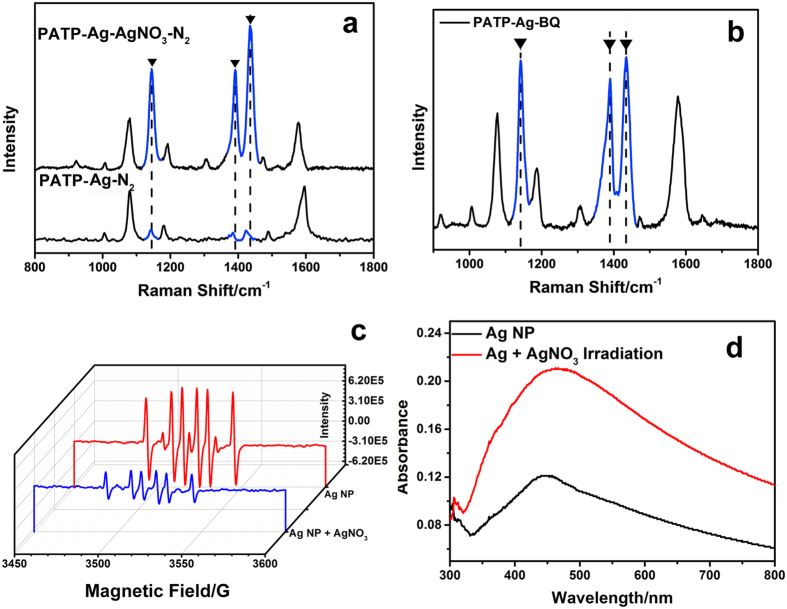
(**a**) SERS signals of PATP on Ag and Ag-AgNO_3_ substrates in N_2_ atmosphere; (**b**) SERS signals of PATP in the presence of BQ; (**c**) EPR spectra of DMPO on Ag nanoparticles in the absence and presence of AgNO_3_; (**d**) UV-Vis spectra of Ag-AgNO_3_ substrates before and after the light irradiation. The characteristic peaks of 4, 4′-DMAB labelled by ▼.

The above results seem to be inconsistent with the current reports, where the hot electron together with oxygen is generally considered to be responsible for the plasmon-driven oxidation reaction^38^,^39^. To reveal the real role of oxygen and electron during the oxidation of PATP, the reaction was further carried out in N_2_ atmosphere (Fig. 2a), which is significantly prohibited, suggesting O_2_ indeed contributes to the oxidation of PATP. It is highly possible that O_2_ may be reduced by hot electron to •O_2_^−^ with strong oxidation capacity, which further cause the oxidation of PATP. To check this conjecture, a typical •O_2_^−^ capturer, p-benzoquinone (BQ), is further applied in the SERS analysis. However, the synthesis conducted in the atmospheric environment in the presence of BQ does not cause any variation of 4, 4′-DMAB signals (Fig. 2b), excluding the possible effect of •O_2_^−^ on the oxidation of PATP. Intriguingly, when AgNO_3_ is adopted as an electron capturer in N_2_ atmosphere, the oxidation of PATP to 4, 4′-DMAB occurs even in the absence of oxygen. A similar experiment was conducted in the water solution^40^,^41^,^42^,^43^ and the result confirm our suppose (Figure S6). As figure shows, in the water solution, the hole sacrificial agent AO is useful to prohibit the generation of DMAB. And the signal intensity of detected molecule seems weaker for the poor plasmon in the solution.

Electron spin resonance (ESR) is then further adopted to analyse the function of AgNO_3_ in the oxidation of PATP by using 5, 5-dimethyl-1-pyrroline N-oxide (DMPO) as the indicator of •O_2_^−^. It is obvious that the intense signal appears on Ag nanoparticle layer under the laser irradiation, but the presence of AgNO_3_ causes the decreased peak intensity of DMPO-•O_2_^−^ (Fig. 2c), which thus proves two facts as follows. First, O_2_ adsorbed on the surface of Ag nanoparticle layer can actually be reduced by SPR-derived electron. However, the presence of superoxide radical has no effect on the oxidation of PATP; Second, hot electrons can be indeed captured by AgNO_3_ according to the retarded formation of •O_2_^−^. Based on these two facts, the oxidation of PATP in the absence of O_2_ should be attributed to the improved concentration of holes due to the capture of electron by AgNO_3_ (eqs 1–3, SI).

Generally, the SPR-derived hole-electron exciton from Ag nanoparticle layer resides in the Fermi energy level, which is harder to be separated than that formed from semiconductor^44^,^45^,^46^,^47^,^48^,^49^. Since the hole has been revealed as the exclusively active species for the oxidation of PATP instead of O-containing oxidants, the retarded oxidation from PATP to 4, 4′-DMAB in the absence of O_2_ should be attributed to the inefficient separation of hole from electron. As such, molecular O_2_ should actually function as an electron capturer, which consumes electrons and produce enough holes to initiate the oxidation of PATP (eq 4, SI). Moreover, it is noted the plasmonic absorption of Ag nanoparticle layer in the presence of AgNO_3_ (Ag-AgNO_3_) is enhanced under UV-light irradiation (500 W Xe light, Fig. 2d), implying a possible reduction of AgNO_3_ to Ag during the SERS analysis. To understand the influence of enhanced plasmon resonance intensity on the reaction, the SERS of PATP on the pre-irradiated Ag-AgNO_3_ assembled nanoparticle layer has been further investigated in N_2_ atmosphere. The signal intensity attributed to 4, 4′-DMAB seems too low to be detected (Figure S7), thus excluding the contribution from improved plasmon resonance intensity to the conversion efficiency.

**Figure 3:**
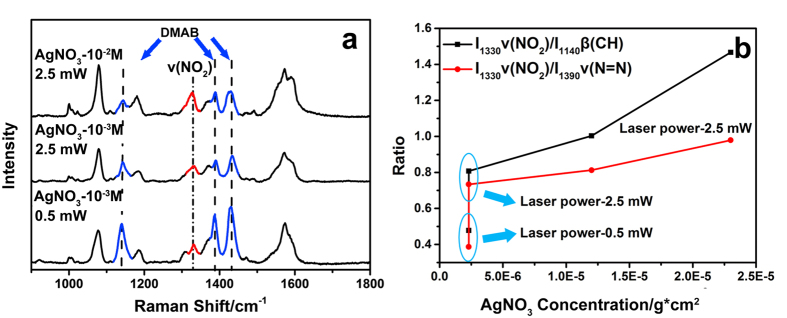
(**a**) SERS signals of PATP on Ag-AgNO_3_ substrates under the irradiation of 532 nm laser light; (**b**) Relation between the density of AgNO_3_ and the composition ratio of PNTP/4, 4′-DMAB.

Furthermore, it is found that when both of AgNO_3_ and atmospheric O_2_ are present, PATP is unprecedentedly transformed into a mixture of PNTP and 4, 4′-DMAB within 10 s as characterized by the appearance of ν (NO_2_) peak at ca. 1330 cm^−1^ (Fig. 3a), demonstrating the oxidation efficiency can be enhanced by improving the separation degree of hole-electron exciton. Both of AgNO_3_ and O_2_ should be involved in the oxidation of 4, 4′-DMAB to PNTP since no PNTP can be produced when either of them is absent. The influence of AgNO_3_ density (ρ_Ag_) on the conversion efficiency was further investigated. It is found from Fig. 3a that 4, 4′-DMAB is the dominant product at a laser power of 0.5 mW and ρ_Ag_ of 2.3^*^10^−6 ^g/cm^2^, as evidenced by the strong peaks at 1440, 1380 and 1140 cm^−1^ from 4, 4′-DMAB and a weak peak at 1330 cm^−1^ due to the ν(NO_2_) of PNTP. The peaks of 4, 4′-DMAB decreases when the laser power and ρ_Ag_ increase to 2.5 mW and 2.3*10^−5 ^g/cm^2^, along with an increasing intensity of ν (NO_2_) peak. The intensity ratio between peaks at 1330 and 1390 cm^−1^ (I_1330_ν(NO_2_)/I_1390_ν(N = N)) was plotted to more clearly demonstrate the co-effect of AgNO_3_ and laser power (Fig. 3a, red line). The ratio of I_1330_ν(NO_2_)/I_1140_β(CH) was also plotted and used as a reference (Fig. 3b, black line). The I_1330_ν(NO_2_)/I_1390_ν(N = N) and I_1330_ν(NO_2_)/I_1140_β(CH) obtained at 2.5 mW are almost doubled compared with those formed at 0.5 mW when ρ_Ag_ is 2.3*10^−6 ^g/cm^2^, which are further improved for ca. 30% and 80% when ρ_Ag_ is increased to 2.3*10^−5 ^g/cm^2^. A higher ρ_Ag_ leads to the decreased intensity of both 4, 4′-DMAB and PNTP (not shown), implying the shielding of Ag nanoparticle layer by overmuch addition of AgNO_3_. The actual composition of PNTP should be higher as valued from the peak intensity ratio between the a_1_ and a_g_ modes since the intensities of the a_g_ modes of 4, 4′-DMAB are significantly stronger than those of the a_1_ modes of PNTP^50^, where a small amount of 4, 4′-DMAB may already produce observable signal in SERS spectra. What’s more, to investigate the role of laser power and exposure time^51^, Ag-AgNO_3_ substrate was taken to detect PATP under 0.5 and 0.25 mW with different exposure times as Figure S8. The laser is both used for the light source of the SPR reaction and SERS analysis. The decreasing of the laser power decreases the reaction efficiency and so does the SERS sensitivity. We further investigated the reaction under 0.25 mW irradiation, where the Raman intensity is decreased without obvious variation of the reaction efficiency. The variation of the irradiation time to 5 s or 3 min does not obviously cause the change of the reaction process. However, when the exposure time extends to 10 min, the sample seems to be destroyed by the strong laser power, weakening the signal of the PNTP and DMAB on the substrate.

**Figure 4:**
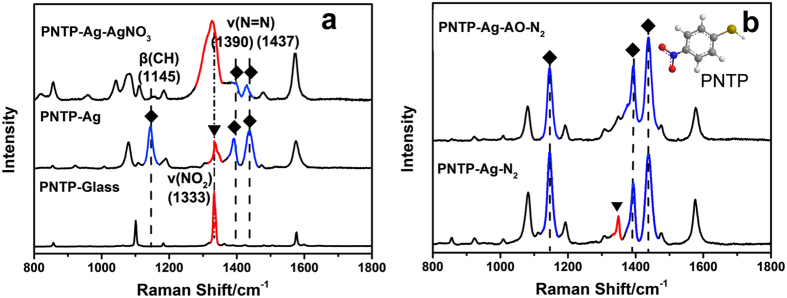
(**a**) Raman spectra of PNTP on the glass slide, SERS signals of PNTP on assemble Ag layer in the absence and presence of AgNO_3_. (**b**) SERS signals of PNTP on the Ag and Ag-AO substrates in N_2_ atmosphere.

As a further check for the feasibility of radical-capture strategy to the *in-situ* SERS analysis of other SPR catalytic reactions, the SPR-catalysed reduction of PNTP, another typical reaction model has been further adopted here^31^. The results shown in Fig. 4a indicate the reduction of PNTP is retarded when AgNO_3_ is present, accordant with the commonly-accepted understanding about the function of electrons in the SPR-catalysed reduction reaction^3^,^4^. On the contrary, the reduction can be promoted by conducting the reaction in N_2_ atmosphere (Fig. 4b), which should be attributed to the eliminated consumption of electron by molecular O_2_. Even more, a higher reduction degree of PNTP is achieved when AO is adopted to improve the electron concentration through consuming more holes (Fig. 4b).

## Conclusions

In summary, we have explored the mechanism of SPR-catalysed reaction by a capturer-assisted SERS strategy using the oxidation of PATP on Ag nanoparticle layer as the model reaction. The adoption of AO and AgNO_3_ as the capturers for hole and electron effectively leads to the separation of SPR-derived hot holes and electrons. The hot hole is directly responsible for the oxidation of PATP to 4, 4′-DMAB. The oxidation of PATP is prohibited in N_2_ atmosphere but occurs when AgNO_3_ is further present. O_2_ plays the role as an electron capturer in promoting the separation of hole-electron. The oxidation of PATP to PNTP has been unprecedentedly achieved in the atmospheric environment when the reaction is assisted by AgNO_3_. This study provides a novel way to deeply understand the mechanism of plasmon-related photocatalysis and photochemical reactions, which is expected to substantially push the development of SPR-induced green synthesis forward through rational and scientific design.

## Method

### Experimental Section

#### Preparation of Ag Nanoparticles with Diameter of ca. 50 nm

The preparation of Ag nanoparticles is adopted from a previously reported method^52^. In a typical synthesis, 85.0 mg of PVP was dispersed into 20.0 mL of water under magnetic stirring. After the complete dissolution of PVP, 85.0 mg of AgNO_3_ and 200 μL of 5.0 M NaCl were successively added under rapid stirring. The mixture was kept stirring in the dark for 15 min to form AgCl colloid. The freshly prepared AgCl colloid was then used as the precursor for Ag nanoparticles to achieve a size-controlled synthesis. First, 20.0 mL of 50.0 mM ascorbic acid was added to 2.5 mL of 0.5 M NaOH under magnetic stirring, and 2.5 mL of freshly prepared AgCl colloid is also added. The mixture was then stirred for 2 h in the dark. The products are collected by centrifugation and washed with water and kept in an ethanol solution.

#### Preparation of assembled Ag nanoparticle layer

Assembled Ag nanoparticle layer was spin-coated from the ethanol solution of Ag nanoparticles (1.0 mL, 0.05 M) and used for SEM, AFM and UV-Vis diffuse reflectance analyses. PNTP or PATP (200 μL, 10^−2 ^M) was dispersed on Ag nanoparticle layer by premixing the molecules with the Ag ethanol solution and the mixture was spin-coated using the above procedure. For the study in the presence of capturing agent, 200 μL ethanol solution of AgNO_3_, AO, CH_3_OH and BQ (10^−3^–10^−2 ^M) was mixed with 200 μL PNTP or PATP solution (10^−2 ^M) before the spin-coating process.

#### Characterization

Scanning electron microscopy (SEM) analysis was performed using a TESCAN nova III scanning electron microscope. Transmission electron microscopy (TEM) analysis was performed using a JEOL 2100 LaB6 TEM, at a 200 kV accelerating voltage. Raman spectra were recorded on a Renishaw inVia-Reflex Raman microprobe system equipped with Peltier charge-coupled device (CCD) detectors and a Leica microscope. Spectra were collected from the nanoparticle layer with an accumulation time of 10 s. Lasers with wavelength of 532 nm and 785 nm were used as the excitation light source, and a 50× objective with a numerical aperture (NA) of 0.75 was used to get the laser spot diameter of ~1 μm. The electron spin resonance (ESR) technique (with DMPO) was used to detect the radical species over the catalyst on a Bruker EMX-8/2.7 spectrometer. DMPO was added to the suspension system before testing, and then the system was irradiated by visible light using a Xenon lamp. Electron spin resonance (ESR) technique is a very powerful and sensitive method for the characterization of the electronic structures of materials with unpaired electrons. By investigating the resonance line can obtain the information about status of the unpaired electrons in radical and its surrounding environmental, thereby obtaining information about the structure and chemical bonding of the substance, in order to identify the different types of free radicals and their levels.

## Additional Information

**How to cite this article**: Yan, X. *et al*. Surface-Enhanced Raman Spectroscopy Assisted by Radical Capturer for Tracking of Plasmon-Driven Redox Reaction. *Sci. Rep.* **6**, 30193; doi: 10.1038/srep30193 (2016).

## Supplementary Material

Supplementary Information
