# Supplementary material for: Surface-Enhanced Raman Spectroscopy Assisted by Radical Capturer for Tracking of Plasmon-Driven Redox Reaction
Source: Sci Rep. 2016 Jul 22;6:30193. doi: 10.1038/srep30193 (PMC4957100; doi:10.1038/srep30193)
Supplement: Supplementary Information [file srep30193-s1.docx]

Surface-Enhanced Raman Spectroscopy Assisted by Radical Capturer for Tracking of Plasmon-Driven Redox Reaction

Xuefeng Yan, Lingzhi Wang,* Xianjun Tan, Baozhu Tian, Jinlong Zhang*

Key Lab for Advanced Materials and Institute of Fine Chemicals, East China University of Science and Technology, Meilong Road 130, Xuhui District, Shanghai, 200237, P. R. China

**Chemical:**

Sodium chloride, Sodium bicarbonate, ammonium oxalate (AO) and t-butanol (TBA) were purchased from Shanghai Lingfeng Chemical Reagent Co. Ltd without further purification. Silver nitrate (AgNO3) and polyvinyl pyrrolidone K-30, Sodium hydroxide and L-ascorbic acid were purchased from Shanghai Chemical Reagent. p-nitrothiophenol and p-aminothiophenol were purchased from Aladdin. Ultrapure water (=18.0 MΩ) purified using the Milipore Milli-Q gradient system. 5, 5-dimethyl-1-pyrroline N-oxide (DMPO) was purchased from J&K scientific Ltd.


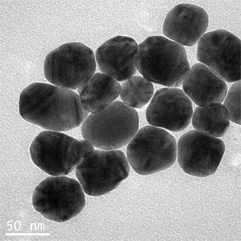


Figure S1. TEM image of Ag nanoparticles.


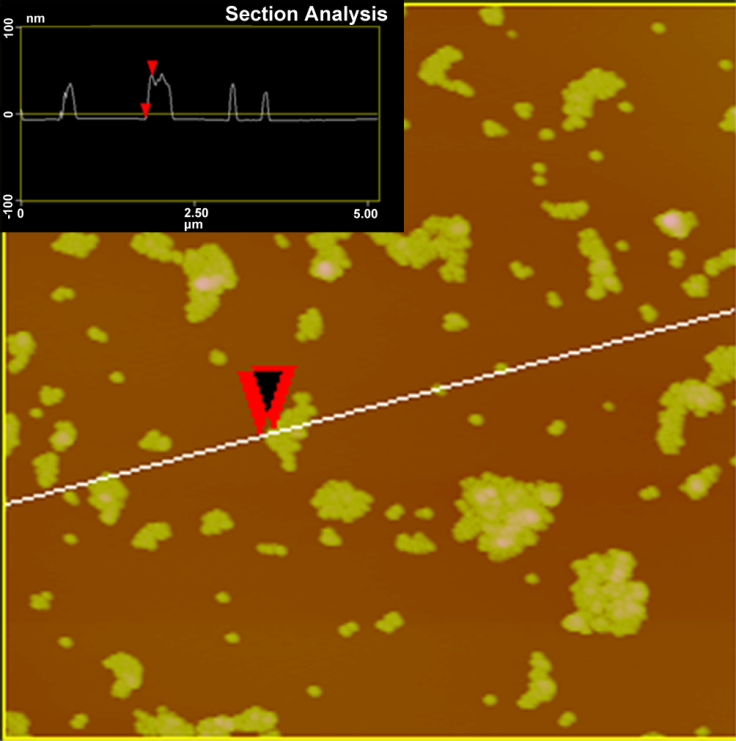


Figure S2. AFM image of Ag film.


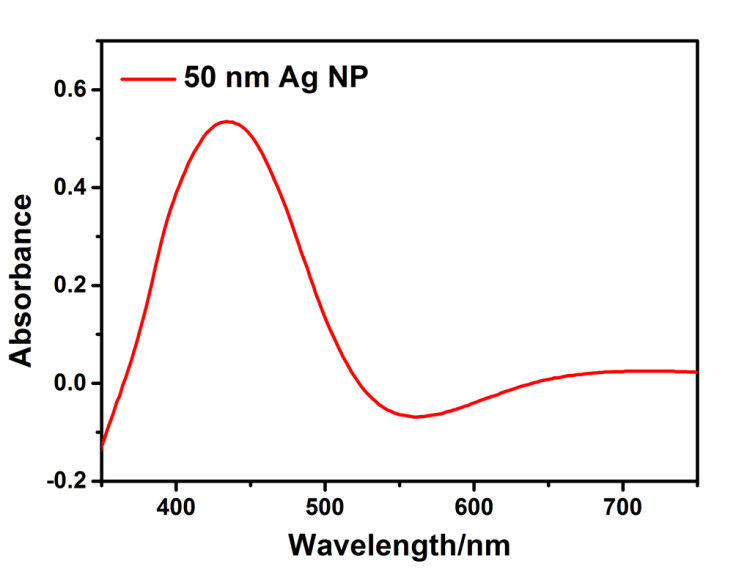


Figure S3. UV-vis spectrum of Ag nanoparicle.


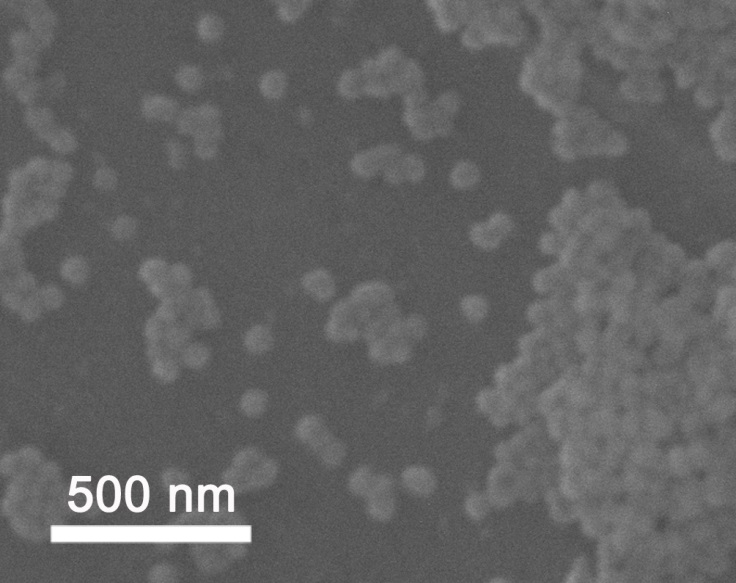


Figure S4. SEM of assemble Ag nanoparticles layer.


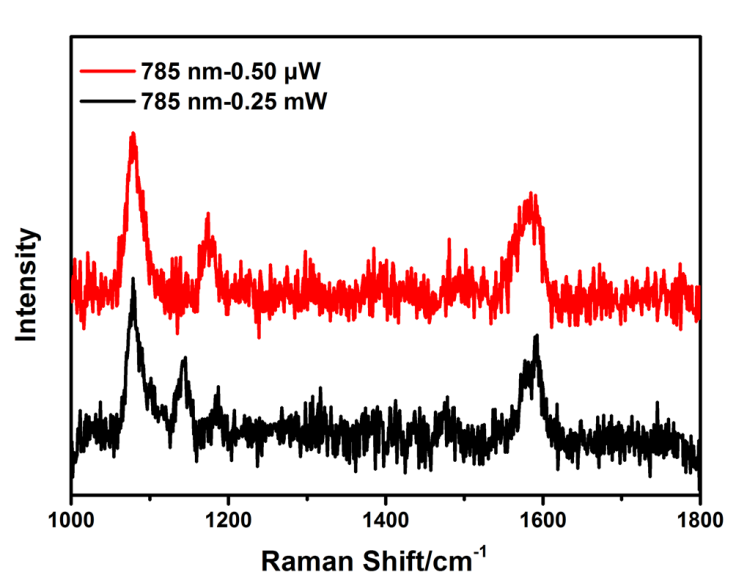


Figure S5. SERS signal of PATP on Ag substrate on the irradiation of 785 nm with two laser power. The intensity of the peaks have be normalized at 1090 cm^-1^


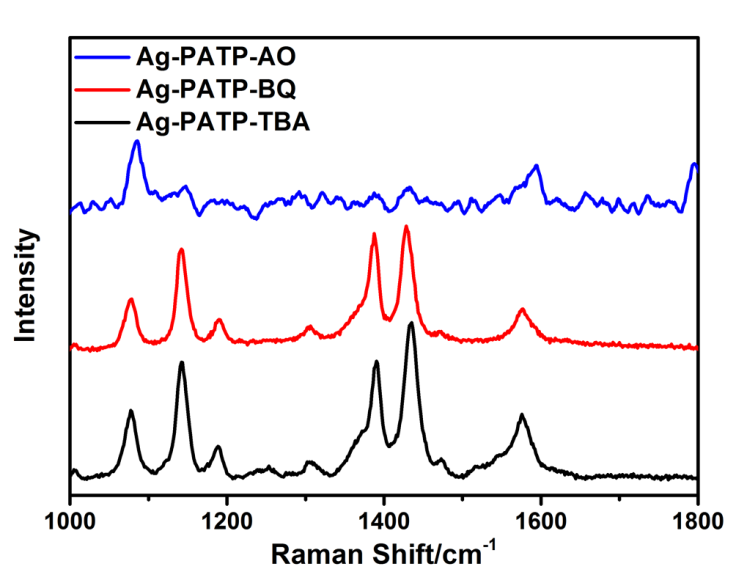


Figure S6. SERS signals of PATP on Ag substrates with different sacrificial agents in the water solution.


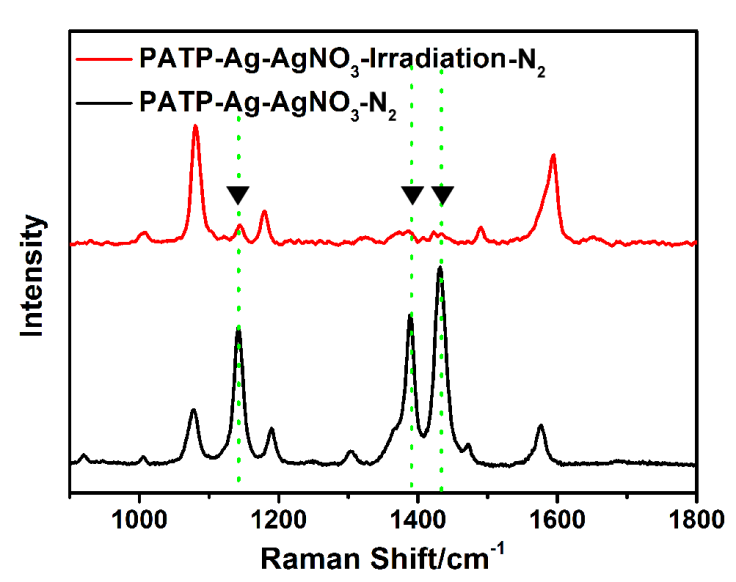


Figure S7. SERS signals of PATP on Ag-AgNO_3_ substrates before and after irradiation in N_2_ atmosphere. The characteristic peaks of 4, 4’-DMAB are labelled by ▼.


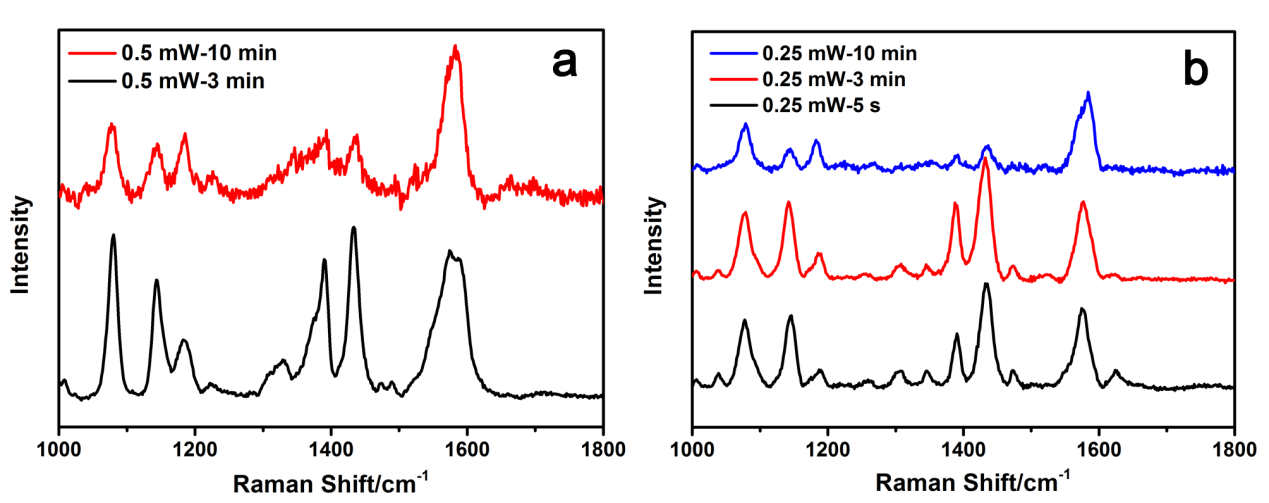


Figure S8. SERS signals of PATP on Ag-AgNO_3_ substrates under the irradiation of 532 nm laser light of 0.5 mW (a) and 0.25 mW (b) and different exposure times.

Equations for the SPR-catalyzed oxidation of PATP accelerated by electron capturers of O_2_ and AgNO_3_.

Ag + hν → Ag* → Ag (e-h^+^); (1)

Ag (e-h^+^) + AgNO_3_ → Ag (h^+^) + Ag; (2)

Ag (h^+^) + PATP → DMAB; (3)

Ag (e-h^+^) + O_2_ → Ag (h^+^) + •O_2_^-^; (4)
